# Low RBM3 Protein Expression Correlates with Clinical Stage, Prognostic Classification and Increased Risk of Treatment Failure in Testicular Non-Seminomatous Germ Cell Cancer

**DOI:** 10.1371/journal.pone.0121300

**Published:** 2015-03-26

**Authors:** Sven-Erik Olofsson, Björn Nodin, Alexander Gaber, Jakob Eberhard, Mathias Uhlén, Karin Jirström, Mats Jerkeman

**Affiliations:** 1 Department of Clinical Sciences, Division of Oncology and Pathology, Lund University, Skåne University Hospital, Lund, Sweden; 2 Science for Life Laboratory and School of Biotechnology, AlbaNova University Center, Royal Institute of Technology, Stockholm, Sweden; 3 School of Biotechnology, AlbaNova University Center, Royal Institute of Technology, Stockholm, Sweden; Taipei Medical University, TAIWAN

## Abstract

**Background:**

Expression of the RNA-binding motif protein 3 (RBM3) has been shown to correlate with favourable clinicopathological parameters and prognosis in several cancer diseases. The aim of this study was to examine the expression and prognostic ability of RBM3 in patients with testicular non-seminomatous germ cell tumours (NSGCT).

**Patients and Methods:**

Immunohistochemical RBM3 expression was analysed in tissue microarrays with tumours from 206 patients. Chi-square test was applied to analyze associations between RBM3 expression and clinicopathological parameters. Kaplan-Meier analysis was used to assess the impact of RBM3 expression on cancer-specific survival (CSS) and failure-free survival (FFS). Cox regression proportional hazards models were used to estimate the relative risk for failure.

**Results:**

In the entire cohort, there was a significant association between clinical stage (*p=0*.*044*) and RBM3 expression. Weak RBM3 expression correlated with a significantly reduced FFS [79.3% versus 90.4% (*p=0*.*019*)] and CSS [87.5% versus 97.3% (*p=0*.*047*)]. For patients with metastatic disease (*n = 88*), significant associations were found between RBM3 expression and IGCCC group (*p=0*.*007*). The FFS was significantly inferior for patients with low tumour-specific RBM3 expression [59.3% versus 79.0% (*p=0*.*013*)], and this association remained significant in a multivariable model for patients with metastatic disease (HR=3.67; 95% CI 1.14, 11.89).

**Conclusion:**

Low RBM3 expression is an independent predictor of treatment failure in metastatic NSGCT, in relation to the prognostic factors included in the International Germ Cell Consensus Classification (IGCCC). These findings suggest that RBM3 may be a potential biomarker for treatment stratification in patients with metastatic non-seminomatous germ cell tumours, and therefore merit further validation.

## Introduction

Since the introduction of cisplatin into the treatment of testicular cancer (TC), more than 95% of patients are cured. For patients without any evidence of metastatic disease, i.e. clinical stage I (CS I), survival approaches 100% [1, 2]. The International Germ Cell Consensus Classification (IGCCC) for non-seminomatous germ cell tumours (NSGCT)[3] is used to classify patients with metastatic disease into three groups with good, intermediate or poor prognosis, based on clinical parameters present prior to therapy (S1 Table). In a meta-analysis, performed in 2005, 5-year survival estimates for patients with good prognosis were 94%, intermediate prognosis 83% and 71% in the poor prognosis group[4]. The majority of patients are young at diagnosis and it is therefore important to avoid long term morbidity that may result from TC treatment. Hence, although certain clinicopathological factors have proven to be of value to guide treatment, there is a clear need for additional prognostic markers to better discriminate between patients with high-risk and low-risk disease[5]. In CS I patients, the risk factors for occult metastatic disease are vascular/lymphatic invasion, proliferation rate > 70% and more than 50% embryonal carcinoma[6]. Immunohistochemical assessment of tumor proliferation using MIB-1 (Ki-67 receptor) can be used to identify patients in CS I with low risk for relapse. The MIB-1 antibody can also be used in the diagnosis of NSGCT[6].

Low expression of RNA binding motif protein 3 (RBM3) has been demonstrated to correlate with a worse prognosis in several major cancer forms, e.g. breast, ovarian, prostate, colorectal, bladder cancer and malignant melanoma[7–12]. RBM3 expression has also been demonstrated to correlate with sensitivity to platinum-based chemotherapy in ovarian cancer in vivo and in vitro[8, 13]. In gonads, RBM3 has been found to be expressed in Sertoli cells, but not germ cells, of seminiferous tubules[14]. The aim of the present study was to examine the expression and prognostic significance of RBM3 in NSGCT. In this paper, we have not examined the diagnostic impact of RBM3 expression.

## Materials and Methods

### Patients

All adult patients diagnosed with testicular NSGCT in the Southern Sweden Health Region between July 1, 1995 and December 31, 2007, in whom clinical data were available, were included in this study. The survival status of all patients was checked against the national population registry in 2012. The patients were treated according to the protocols SWENOTECA (Swedish-Norwegian Testicular Cancer Group) IV (metastatic disease) and SWENOTECA III and VI (non-metastatic disease)[15]. The SWENOTECA database holds detailed information on tumour stage, prognostic classification, treatment, short- as well as long- term treatment toxicity and follow-up data 10 years after diagnosis.

### Ethics Statement

All EU and national regulations and requirements for handling human samples have been fully complied with during the conduct of this project; i.e. decision no. 1110/94/EC of the European Parliament and of the Council (OJL126 18,5,94), the Helsinki Declaration on ethical principles for medical research involving human subjects, and the EU Council Convention on human rights and Biomedicine. The study was approved of by the Ethics committee of Lund University (ref nr 386/99), whereby the committee waived the need for consent other than by the option to opt out.

### Materials

Patients were identified in the SWENOTECA database and the corresponding slides and formalin-fixed paraffin-embedded tissue blocks were retrieved from the pathology archives. Prior to tissue micro array (TMA) construction, all available haematoxylin and eosin stained slides from each case were re-evaluated by a board-certified pathologist (KJ). The total number of histological subtypes as well as their estimated proportions was denoted. Irrespective of the number of histological components, a standard set of 4x1mm cores were taken from each invasive tumour in a proportional fashion, covering up to 3 different components. In addition, 2x1 mm cores were sampled from areas with intratubular germ cell neoplasia (ITGCN) from 127 cases and adjacent, benign-appearing testis from 49 cases. A semi-automated arraying device was used (TMArrayer; Pathology Devices, Inc, Westminster, MD, USA). For immunohistochemical analysis of RBM3, 4 μm TMA-sections were automatically pre-treated (deparaffinization, rehydration and epitope retrieval) using the PT-link system (DAKO, Copenhagen, Denmark) and then stained in a Techmate 500 (DAKO, Copenhagen, Denmark) with the mouse monoclonal RBM3 antibody, clone CL0296, product ID AMAb90655, Atlas Antibodies AB, Stockholm, Sweden (formely known as AAb030038, Atlas Antibodies, Stockholm, Sweden) diluted 1:5000. The antibody has been validated in previous studies[8, 10]. For assessment of nuclear RBM3 expression, both the fraction of positive cells and staining intensity were taken into account for each tissue core using a semiquantitative scoring system, whereby the estimated percentage of cells with nuclear RBM3 expression was recorded as nuclear fraction (NF) and categorized into four groups, namely 0 (0–1%), 1 (2–25%), 2 (26–50%), 3 (51–75%), 4 (>75%) and the nuclear staining intensity (NI) denoted as 0 = negative, 1 = mild, 2 = intermediate and 3 = strong intensity. A combined nuclear score (NS) of NFxNI, which had a range of 0 to 12, was then constructed. Cytoplasmic staining intensity was denoted as 0 = negative, 1 = mild and 2 = moderate-strong, and the fraction of positive cells not taken into account. The staining was evaluated by two independent observers (KJ, SEO) who were blinded to clinical and outcome data.

### Endpoints and statistical methods

RBM3 expression (intensity × fraction) was dichotomized into weak vs strong using classification and regression tree (CRT) analysis[16]. Chi-square and Mann-Whitney U tests were used for comparison between clinicopathological characteristics and RBM3 expression. Treatment failure was defined as relapse after treatment for metastatic disease, relapse for patients in CS I receiving adjuvant chemotherapy, finding of active cancer at surgery post chemotherapy or death from NSGCT. A contralateral tumor was not considered a relapse. Failure-free survival (FFS) was defined as time from orchiectomy to treatment failure or otherwise censored at last follow-up (FU) or death from other causes. Overall survival (OS) was defined as time from orchiectomy to death from any causes or otherwise censored at last FU. Cancer specific survival (CSS) was defined as time from orchiectomy to death from NSGCT or treatment or otherwise censored at last FU or death from other causes. Time to relapse was defined, for disease-free patients, as time from end of treatment to relapse and censored at last FU or death from other causes. Survival and cumulative incidence curves for the above endpoints were estimated using the Kaplan-Meier method and log-rank tests were used for comparisons. Cox regression proportional hazards models were used to estimate the relative risk for failure in both uni- and multivariable analysis. A p-value of 0.05 was considered significant and all tests were two-sided. All statistical analyses were performed using IBM SPSS Statistics version 20.0 (SPSS Inc., Chicago, IL, USA).

## Results

### Patient characteristics

Between July 1, 1995 and December 31, 2007 a total number of 300 adult patients were diagnosed with testicular NSGCT in the Southern Sweden Health Region. In 219 patients, clinical data were available in the SWENOTECA database at the time of data extraction.

Thirteen (6%) cases were excluded from the study; four patients who started chemotherapy before orchiectomy, three cases with missing tissue blocks and six cases with an insufficient amount of tissue available for analysis. Of the 206 included patients, 118 (57%) had clinical stage 1 (CSI) disease and 88 (43%) metastatic disease. Patient characteristics, therapy-related data, and survival data are presented in Table 1. In patients with metastatic disease at diagnosis (CSII-IV and elevated marker only (Mk+)), 73% were classified as having good prognosis, 15% intermediate prognosis and 12% poor prognosis according to IGCCC. The median FU for surviving patients (*n = 193*) was 76 months (range 3–167). The 5-year OS was 96.0% for all patients. Thirteen patients died during follow-up, six due to progressive disease and two due to treatment. Five patients died of other causes without evidence of NSGCT. There were 14 relapses, six in patients with metastatic disease in complete remission after chemotherapy and surgery (*n =* 61, 69%) and eight in patients with CS I disease whereof three after adjuvant chemotherapy. Twenty patients with CS > I disease had treatment failure, 14 with vital cancer in the specimen after surgery, five relapses and one died due to progression of disease. Three patients with CS I disease had a relapse after adjuvant treatment whereof one later died in testicular cancer.

**Table 1:** Characteristics, treatment, relapse and survival of 206 patients with non-seminomatous testicular cancer.

			Good	Intermediate	Poor
	All	CS I	prognosis	prognosis	prognosis
	*N* (%)	*N* (%)	*N* (%)	*N* (%)	*N* (%)
*N* (%)	206 (100)	118 (57)	64 (31)	13 (6)	11 (5)
**Age** median (range)	29 (18–68)	29 (18–68)	28 (19–58)	28 (20–42)	29 (22–48)
16–34 *N* (%)	151 (73)	83 (70)	50 (78)	11 (85)	7 (64)
35-> *N* (%)	55 (27)	35 (30)	14 (22)	2 (15)	4 (36)
**Clinical stage** ^‡^					
CS I *N* (%)	118 (57)	118 (100)			
Mk+ *N* (%)	6 (3)		5 (8)	0	1 (10)
CS II *N* (%)	45 (22)		38 (59)	6 (46)	0
CS III *N* (%)	3 (3)		2 (3)	1 (8)	0
CS IV *N* (%)	34 (16)		19 (30)	6 (46)	9 (82)
**Primary treatment**					
CS I adjuvant		85 (72)			
1 regimen *N* (%)	64 (31)		53 (83)	10 (77)	1 (9)
2 regimens *N* (%)	21 (10)		11 (17)	2 (15)	8 (73)
> = 3 regimens *N* (%)	4 (2)		1 (2)	1 (8)	2 (18)
HDCHT *N* (%)	6 (3)		0 (0)	0 (0)	6 (54)
Surgery *N* (%)	61 (30)		39 (61)	13 (100)	9 (82)
PAD-cancer *N* (%)	15 (25)		10 (16)	0 (0)	5 (56)
**Relapse occurrence *N* (%)**	14 (7)	8 (7)	3 (5)	1 (8)	2 (18)
**Dead *N* (%)**	13 (6)	7 (6)	2 (3)	1 (8)	3 (27)
Testicular cancer *N* (%)	6 (46)	1 (14)	1 (50)	1 (100)	3 (100)
Treatment *N* (%)	2 (15)	1 (14)	1 (50)	0	0
Other *N* (%)	5 (38)	5 (71)	0	0	0
**Treatment failure** ^↑^ ***N* (%)**	23 (11)	3 (2)	13 (20)	1 (8)	6 (54)
**Survival**					
FU (m) median (range)	76 (3–167**)**	75 (3–142)	72 (9–167)	98 (57–125)	103 (50–134)
5-year OS %	96.0	98.2	96.8	92.3	72.7
5-year CSS %	96.6	99.1	96.8	92.3	72.7
5-year FFS %	89.6	97.4	80.3	92.3	34.1

^‡^ According to MRC[17].

^↑^ Relapse after treatment for metastatic disease, finding of active cancer at surgery post chemotherapy, or death from NSGCT.

Abbreviations: AFP, α-fetoprotein; β-HCG, β–human chorionic gonadotropin; HDCT, high-dose chemotherapy; OS, overall survival; CSS, cancer-specific survival; FFS, failure-free survival.

### RBM3 expression

**Fig 1:**
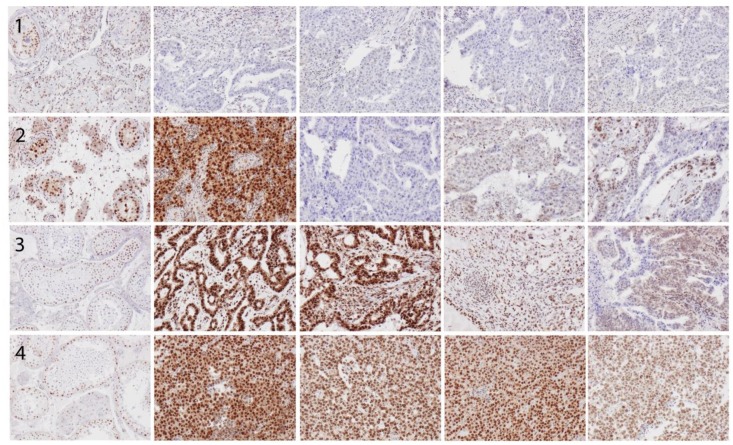
Sample images (10X magnification) of immunohistochemical RBM3 expression in different tissue entities from four cases. Case 1—column 1: atrophic testis; column 2–4: embryonal carcinoma with negative RBM3 expression (note positive stromal lymphocytes). Case 2—column 1: intratubular germ cell neoplasia (ITGCN); column 2–4: embryonal carcinoma with <10% seminoma (column 2) with best score 12 and worst score 2. Case 3—column 1 normal testis; column 2–4: tumour with components of embryonal carcinoma, immature teratoma and yolk sac tumour with best score 12 and worst score 8. Case 4—column 1 normal testis; column 2–4: tumour with predominant seminomatous histology (~80%) admixed with teratoma (not represented in the TMA), best score 12 and worst score 8.

Sample immunohistochemical images are shown in Fig 1.

In normal testis, RBM3 was expressed in weak to moderate intensity in spermatogonia and negative in spermatocytes. In intratubular germ cell neoplasia (ITGCN), RBM3 was expressed with strong intensity in the vast majority of neoplastic cells. In testicular germ cell tumor (TGCT), RBM3 was expressed in various intensities and fractions, mainly in the nucleus but also in the cytoplasm, with a particularly strong expression in seminomatous components.

**Fig 2:**
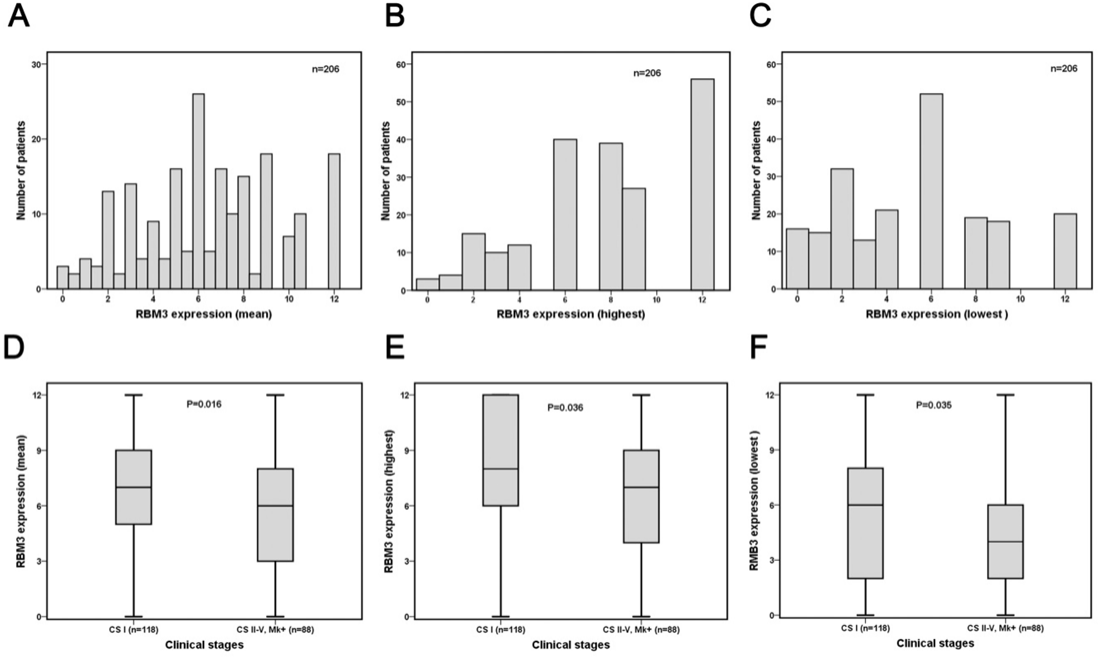
RBM3 staining distribution in non-seminomatous germ cell tumour (NSGCT) denoted as nuclear score (fraction × intensity). (A) mean score, (B) highest score and (C) lowest score. RBM3 staining distribution according to CS I and CS > 1 on (D) mean score, (E) highest score and (F) lowest score. Boxplots shows the five statistics (minimum, first quartile, median, third quartile, and maximum). P-values refer to Mann Whitney U test for comparison of medians.

**Fig 3:**
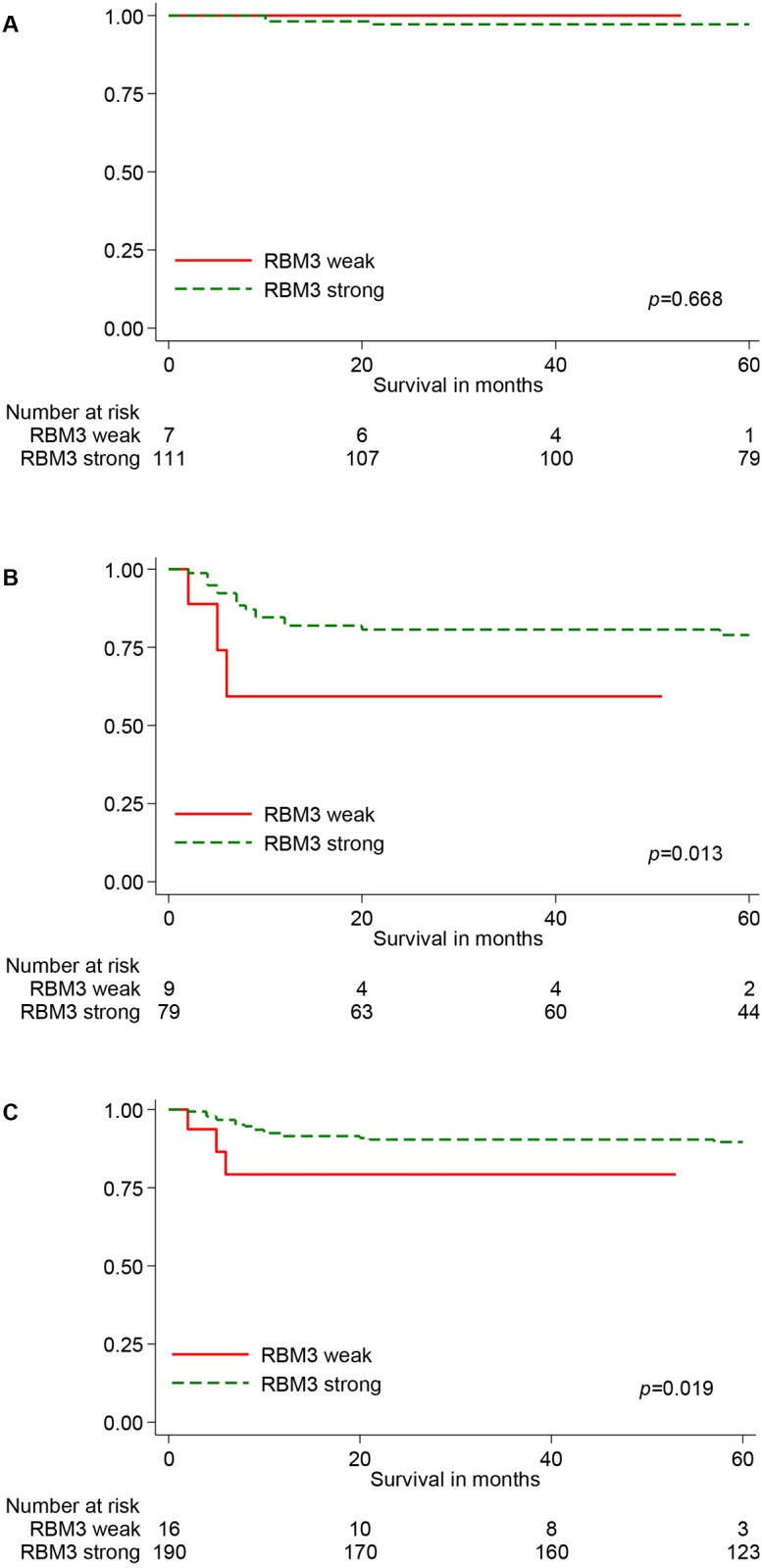
Failure-free survival of patients with non-seminomatous testicular cancer according to RBM3 expression. (A) 118 patients with clinical stage 1, (B) 88 patients with metastatic disease and (C) 206 patients with clinical stage I to IV and Mk+.

The distribution of RBM3 expression for the mean, highest and lowest score is shown in Fig 2. There was a significant difference in mean score of RBM3 expression between tumours from patients in CS I and CS II-IV, Mk+ in all three categories (Fig 2D–2F). The lowest score showed the best correlation to clinicopathological parameters (data not shown)., and has therefore been used in the following statistical analyses CRT analysis suggested a cutoff point at NS > 0.5 to determine the optimal prognostic impact of RBM3 expression on FFS and NS > 2.5 for CSS (S1 Fig). Low RBM3 expression was associated with a significantly worse FFS [79.3% versus 90.4% (*p = 0*.*019*)] (Fig 3C and S2 Table) and CSS [87.5% versus 97.3% (*p = 0*.*047*)] (S2 Table). In the entire cohort, low RBM3 expression (NS < = 0.5) was observed in 16 (7.8%) cases and there was a significant association between clinical stage (*p = 0*.*044*) and RBM3 expression (Table 2). No significant associations were found between age, CS I vs >CS I and RBM3 expression. Cytoplasmic RBM3 expression was not associated with any clinicopathological factors.

**Table 2:** Association between clinicopathological characteristics in 206 patients with non-seminomatous testicular cancer and RBM3 expression.

****RBM3 expression****	****Weak**** ^↑^	****Strong**** ^↓^	*****p*****-value****
**Age**			
16–34 *N* (%)	13 (9)	138 (91)	
35-> *N* (%)	3 (6)	52 (94)	0.454
**Clinical stage**			
CS I *N* (%)	7 (6)	111 (94)	
CS II *N* (%)	2 (4)	43 (96)	
CS III *N* (%)	0	3 (100)	
CS IV *N* (%)	7 (21)	27 (79)	
CS Mk+ *N* (%)	0	6 (100)	0.044
**Clinical stage 1 vs >1**			
CS I *N* (%)	7 (6)	111 (94)	
CS >1 *N* (%)	9 (10)	79 (90)	0.255
**Prognostic classification** ^‡^			
Good *N* (%)	5 (8)	59 (92)	
Intermediate *N* (%)	0	13 (100)	
Poor *N* (%)	4 (36)	7 (64)	0.007
**AFP level** *			
Good *N* (%)	8 (10)	71 (90)	
Intermediate *N* (%)	0	6 (100)	
Poor *N* (%)	1 (50)	1 (50)	0.130
**HCG level** *			
Good *N* (%)	6 (8)	67 (92)	
Intermediate *N* (%)	0	7 (100)	
Poor *N* (%)	3 (43)	4 (57)	0.010
**LDH level** *			
Good *N* (%)	5 (7)	68 (93)	
Intermediate *N* (%)	3 (30)	7 (70)	
Poor *N* (%)	0	1 (100)	0.058
**Combined tumor marker status (AFP, HCG, LDH)** * ^‡^			
Good *N* (%)	5 (8)	59 (92)	
Intermediate *N* (%)	0	14 (100)	
Poor *N* (%)	4 (44)	5 (56)	0.001
**NPVM** *			
No *N* (%)	7 (9)	73 (91)	
Yes *N* (%)	2 (24)	6 (75)	0.148

^↑^Nuclear score < = 0.5

^↓^Nuclear score > 0.5

^‡^ According to ICCCGC

*Patients with CS>1

Abbreviations: AFP, α-fetoprotein; β-HCG, β–human chorionic gonadotropin; LDH, lactate dehydrogenase; NPVM, non-pulmonary visceral metastasis.

### Clinical stage I

For patients in CSI there was no significant associations found between RBM3 expression and outcome (Fig 3A and S2 Table).

### Clinical stage II-IV, Mk+

There was a significant association between RBM3 expression and IGCCC (*p = 0*.*007*), combined tumor marker status (*p = 0*.*001*), and with β–human chorionic gonadotropin (HCG) level (*p = 0*.*010*) (Table 2), but not with levels of α-fetoprotein (AFP) (*p = 0*.*127*), lactate dehydrogenase (LDH) (*p = 0*.*088*) or presence of non-pulmonary visceral metastasis (NPVM) (*p = 0*.*148*). The FFS was significantly inferior for cases with low tumour-specific RBM3 expression [59.3% versus 79.0% (*p = 0*.*013*)] (Fig 3B and S2 Table). Regarding CSS, this difference was not significant [88.9% versus 93.6% (*p = 0*.*618*)] (S2 Table). In a multivariable model; including tumor marker level and the presence of NPVM or not; this risk elevation remained significant (HR = 4.35; 95% CI 1.28, 14.13), when tumor markers were analyzed as continuous variables (Table 3). However, when tumor markers were categorized using the cut-points in the IGCCC, only AFP level remained as a significant prognostic marker. When combined with IGCCC groups in a multivariate model, RBM3 expression was not significantly associated with higher risk for treatment failure (HR = 2.62; 95% CI 0.67, 10.31) (Table 4).

**Table 3:** Cox univariate and multivariate analysis of failure free survival according to RBM3 expression in patients with metastatic non-seminomatous testicular cancer.

	*****N*****	****Crude HR****	****Multivariable HR**** *
	****(events)****	****(95% CI)****	****(95% CI)****
**Tumor marker** (continuous)			
AFP	87 (20)	1.00 (1.00, 1.00)	1.00 (1.00, 1.00)
ß-HCG	87 (20)	1.00 (1.00, 1.00)	1.00 (1.00, 1.00)
LDH	84 (20)	1.25 (1.02, 1.55)	1.00 (0.70, 1.44)
**NPVM**			
No	80 (16)	Ref.	Ref.
Yes	8 (4)	3.41 (1.13, 10.29)	4.95 (0.77, 31.82)
**RBM3 expression**			
Strong	79 (16)	Ref.	Ref.
Weak	9 (4)	3.63 (1.21, 10.93)	4.35 (1.28, 14.13)

* Multivariable analysis on tumor marker status and non-pulmonary visceral metastasis.

Tumor markers (AFP, HCG, LDH) as continuous variables.

**Table 4:** Cox univariate and multivariate analysis of failure free survival according to RBM3 expression in patients with metastatic non-seminomatous testicular cancer.

	*N*	****Crude HR****	****Multivariable HR**** *
	****(events)****	****(95% CI)****	****(95% CI)****
**Tumor marker** (categorical)			
AFP good	79 (16)	Ref.	Ref.
AFP intermediate	6 (2)	1.67 (0.38, 7.28)	1.07 (0.14, 8.35)
AFP poor	2 (2)	3.18 (3.50, 89.56)	61.75 (2.91, 1310.77)
ß-HCG good	73 (16)	Ref.	Ref.
ß-HCG intermediate	7 (1)	0.58 (0.08, 4.41)	0.58 (0.07, 4.69)
ß-HCG poor	7 (3)	3.48 (1.00, 12.14)	0.35 (0.02, 6.53)
LDH good	76 (15)	Ref.	Ref.
LDH intermediate	10 (4)	2.65 (0.88, 8.00)	0.19 (0.01, 2.79)
LDH poor	1 (1)	102.47 (6.32, 1662.49)	21.50 (0.43, 1064.19)
**NPVM**			
No	80 (16)	Ref.	Ref.
Yes	8 (4)	3.41 (1.13, 10.29)	17.80 (0.59, 533.53)
**RBM3 expression**			
Strong	79 (16)	Ref.	Ref.
Weak	9 (4)	3.63 (1.21, 10.93)	2.62 (0.67, 10.31)

* Multivariable analysis on tumor marker status and non-pulmonary visceral metastasis.

Tumor markers (AFP, HCG, LDH) as categorical variables. The cut points defined in the IGCCC are used.

## Discussion

Our results demonstrate that RBM3 expression is upregulated in testicular carcinoma in situ (CIS) compared to normal testis and expressed in various fractions and intensities in TGCT, with the highest expression in seminomatous components. In patients with metastatic disease, reduced RBM3 expression correlated significantly with the established prognostic tool (IGCCC), combined tumor marker status and β-HCG level as well as a significantly shorter time to treatment failure. Furthermore, in a multivariable Cox regression model, including the prognostic factors used in the IGCCC model, low RBM3 expression was an independent predictor for treatment failure, when tumor markers (AFP, HCG and LDH) were entered as continuous variables. RBM3 has been shown to be a prognostic biomarker in several cancers, e.g. urothelial cancer[12], breast cancer[7], colorectal cancer[10], prostate cancer[9], esophageal and gastric adenocarcinoma[18] and ovarian cancer[8]. Moreover, RBM3 expression has been demonstrated to correlate with sensitivity to cisplatin treatment in ovarian cancer in vitro*[8]*. In the present series, low expression of RBM3 was a predictor for increased risk of treatment failure in patients with metastatic NSGCT, and one could hypothesize that RBM3 may be a predictor of sensitivity to cisplatin-based treatment also in this type of cancer. However, if RBM3 was only related to cisplatin sensitivity, one would have expected a higher risk of relapse in patients with CS1 and low RBM3, receiving adjuvant BEP, which was not observed.

A proposed mechanism of action for RBM3 is that it may promote tumorigenesis in early stages of cancer by affecting DDR (DNA damage repair) and checkpoint integrity, thereby lowering the threshold for selection of more malignant clones[8, 13] and once an invasive tumour is established, high levels of RBM3 may influence genomic stability and, hence, chemotherapy sensitivity. In other types of tumours, RBM3 expression has been found to be upregulated in preinvasive and cancerous tissues[9, 19, 20]. In the present study, all samples taken from in-situ components showed high RBM3 expression. CIS has shown to be highly sensitive to radiotherapy, that offers definitive cure[5, 21, 22]. A possible hypothesis is that in most testicular germ cell tumours, the DDR system is virtually not activated and intact and thereby may contribute to the sensitivity to chemotherapy agents like platinum and to radiotherapy. Along this line, RBM3 expression was found to be particularly high in components of seminomatous histology, which are among the most sensitive to radiotherapy[22]. However, as RBM3 expression correlated with tumour stage and HCG level, RBM3 may also be related to inhibition of tumour growth and dissemination, through mechanisms still unknown. Further functional studies are needed, and from a clinical perspective, it would be of interest to examine RBM3 expression in metastases after postchemotherapy surgery, where a low RBM3 expression would indicate reduced cisplatin sensitivity.

The IGCCC discriminates between good, intermediate and poor prognosis patients with metastatic testicular cancer. It is based on primary site, tumour marker level and the presence of NPVM or not. The use of this classification is to guide treatment decisions for the individual patient with the intent to optimize survival and minimize toxicity. Frequently, intermediate- and poor-risk patients are pooled together and treated with similar protocols[23, 24]. Therefore, there is a clear need for new biomarkers, for a more accurate identification of those patients gaining from more intensive treatment upfront, such as dose intensification or high-dose chemotherapy (HDCT) with stem cell support. Our data suggest that RBM3 expression may provide additional prognostic information to that of tumor marker level and pattern of metastasis. When combined with the IGCCC in a multivariable model, RBM3 failed to add significant prognostic information. This may be due to the fact that plasma tumour marker levels (HCG, AFP and LDH) are categorical in the IGCCC, and used as continuous variables in our analysis. For the optimal use of RBM3 in a prognostic model, other cut-off limits for plasma tumour markers may need to be applied.

Taken together, our findings suggest that RBM3 may be a potential biomarker for treatment stratification in patients with metastatic non-seminomatous germ cell tumours, and therefore merit further validation.

## Supporting Information

S1 Fig CRT analysis. CRT analysis for (A) failure free survival and (B) cancer-specific survival.
(TIF)

S1 Table IGCCCG prognostic staging system. International Germ Cell Cancer Collaborative Group prognostic staging system for metastatic non-seminomatous germ cell cancer.
(DOCX)

S2 Table 5-year FFS and 5-year CSS by patient characteristics and RBM3 expression. (DOCX)
