# Supplementary figures and images for: Low RBM3 Protein Expression Correlates with Clinical Stage, Prognostic Classification and Increased Risk of Treatment Failure in Testicular Non-Seminomatous Germ Cell Cancer
Source: PLoS One. 2015 Mar 26;10(3):e0121300. doi: 10.1371/journal.pone.0121300 (PMC4374873; doi:10.1371/journal.pone.0121300)

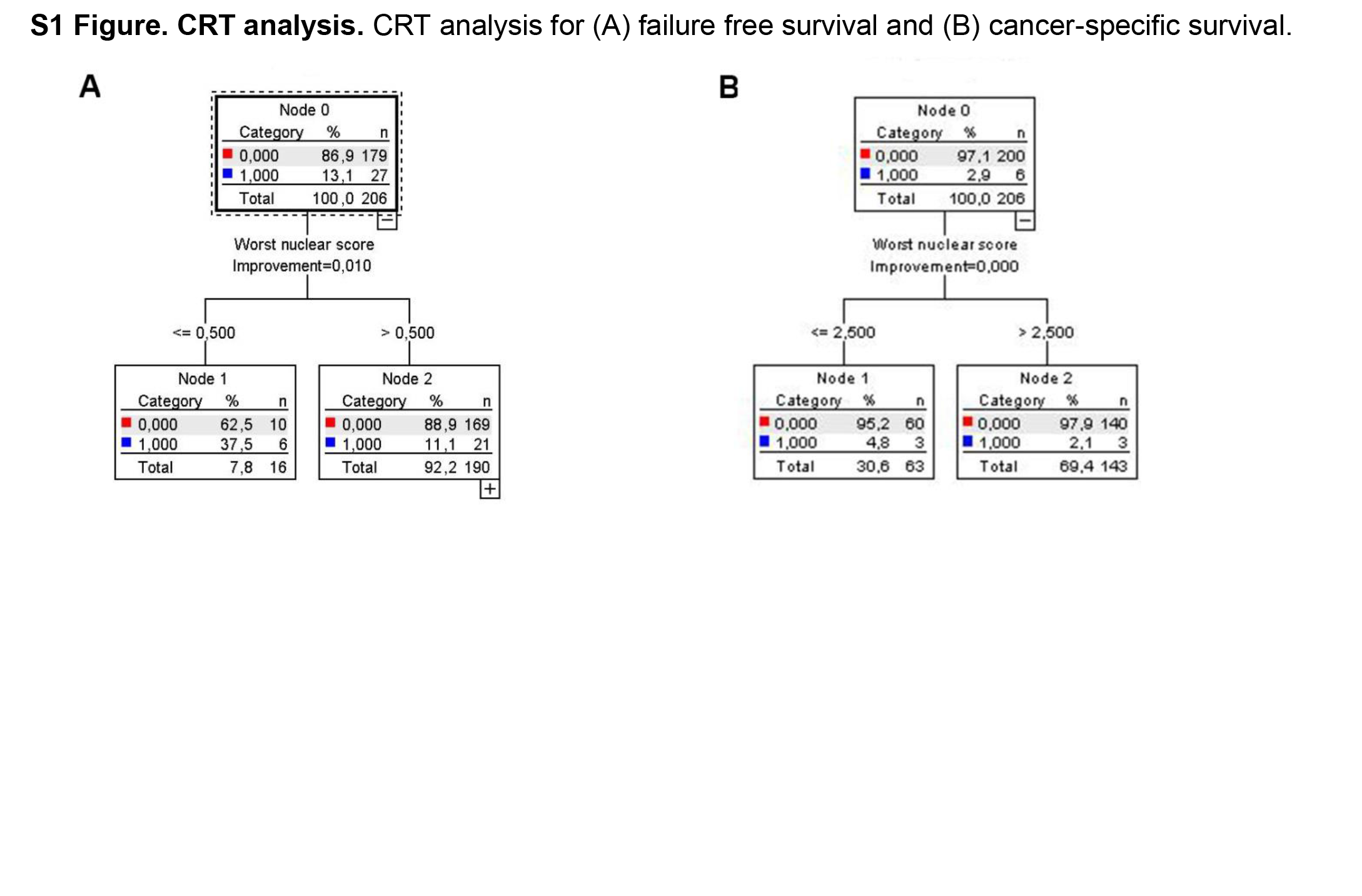

Supplement: S1 Fig — CRT analysis for (A) failure free survival and (B) cancer-specific survival. (TIF) [file pone.0121300.s001.tif]
