# Supplementary material for: Low RBM3 Protein Expression Correlates with Clinical Stage, Prognostic Classification and Increased Risk of Treatment Failure in Testicular Non-Seminomatous Germ Cell Cancer
Source: PLoS One. 2015 Mar 26;10(3):e0121300. doi: 10.1371/journal.pone.0121300 (PMC4374873; doi:10.1371/journal.pone.0121300)
Supplement: S1 Table — International Germ Cell Cancer Collaborative Group prognostic staging system for metastatic non-seminomatous germ cell cancer. (DOCX) [file pone.0121300.s002.docx]

**S1 Table. IGCCCG prognostic staging system.** International Germ Cell Cancer Collaborative Group prognostic staging system for metastatic
 non seminomatous germ cell cancer.

| **Prognostic group** | **Criteria** |
| --- | --- |
| **Good prognosis** | AFP < 1000 ng/ml and β-HCG < 5000 IU/l and LD < 1.5 x ULN and no primary mediastinal tumor and no non-pulmonary visceral metastasis |
| **Intermediate prognosis** | AFP 1000-10000 ng/ml or β-HCG 5000-50000 IU/l or  LD 1.5 -10x ULN and no primary mediastinal tumor and no non-pulmonary visceral metastasis |
| **Poor prognosis** | AFP > 10000 ng/ml or β-HCG > 50000 IU/l or LD > 10 x ULN or primary mediastinal tumor or non-pulmonary visceral metastasis |

Abbreviations: AFP, α-fetoprotein; β-hCG, β-human chorionic gonadotrophin;
 LDH; lactate dehydrogenase.
