# Supplementary material for: Low RBM3 Protein Expression Correlates with Clinical Stage, Prognostic Classification and Increased Risk of Treatment Failure in Testicular Non-Seminomatous Germ Cell Cancer
Source: PLoS One. 2015 Mar 26;10(3):e0121300. doi: 10.1371/journal.pone.0121300 (PMC4374873; doi:10.1371/journal.pone.0121300)
Supplement: S2 Table — (DOCX) [file pone.0121300.s003.docx]

**S2 Table.** **5-year FFS and 5-year CSS by patient characteristics and RBM3 expression.**

| **Characteristics** | **Patients *N* (%)** | **5-year FFS %** | **Log rank *p* value** | **5-year CSS %** | **Log rank *p* value** |
| --- | --- | --- | --- | --- | --- |
| **Age (years)** |  |  |  |  |  |
| <35 | 151 (73) | 89.6 |  | 97.3 |  |
| >=35 | 55 (27) | 88.9 | 0.972 | 94.5 | 0.135 |
|  |  |  |  |  |  |
| **CS 1** | 118 (57) |  |  |  |  |
| Vasc- | 76 (64) | 100 |  | 100 |  |
| Vasc+ | 42 (36) | 94.1 | 0.403 | 97.6 | 0.054 |
|  |  |  |  |  |  |
| **Prognostic index***^‡^ | 88 (43) |  |  |  |  |
| Good | 64 (73) | 80.3 |  | 96.8 |  |
| Intermediate | 13 (15) | 92.3 |  | 92.3 |  |
| Poor | 11 (12) | 34.1 | 0.001 | 72.7 | 0.011 |
|  |  |  |  |  |  |
| **Tumor marker status*** | 87 (42) |  |  |  |  |
| Good | 64 (74) | 80.6 |  | 96.8 |  |
| Intermediate | 14 (16) | 85.7 |  | 92.9 |  |
| Poor | 9 (10) | 29.6 | 0.000 | 66.7 | 0.003 |
|  |  |  |  |  |  |
| **NVPM*** |  |  |  |  |  |
| No | 80 (91) | 80.4 |  | 94.9 |  |
| Yes | 8 (9) | 43.8 | 0.019 | 75.0 | 0.026 |
|  |  |  |  |  |  |
| **RBM3 expression CS I-IV, Mk+** |  |  |  |  |  |
| Weak | 16 (8) | 79.3 |  | 87.5 |  |
| Strong | 190 (92) | 90.4 | 0.019 | 97.3 | 0.047 |
|  |  |  |  |  |  |
| **RBM3 expression CS I** |  |  |  |  |  |
| Weak | 7 (6) | 100 |  | 85.7 |  |
| Strong | 111 (94) | 97.3 | 0.668 | 100 | 0.001 |
|  |  |  |  |  |  |
| **RBM3 expression CS II-IV, Mk+** |  |  |  |  |  |
| Weak | 9 (10) | 59.3 |  | 88.9 |  |
| Strong | 79 (90) | 79.0 | 0.013 | 93.6 | 0.618 |
|  |  |  |  |  |  |

* Patients with CS>1

^‡^ According to ICCCGC
Abbreviations: NPVM , non-pulmonary visceral metastasis.
